# Small bowel obstruction secondary to strangulated obturator hernia with transected ileal segment: A case report

**DOI:** 10.1016/j.ijscr.2025.111098

**Published:** 2025-02-28

**Authors:** Sabin K. Ghimire, Samrat Shrestha, Rahul Jha, Suresh Maharjan, Mecklina Shrestha

**Affiliations:** a National Academy of Medical Sciences, NAMS, Bir Hospital, Department of General Surgery, Kathmandu, Province-3, Nepal; b College of Medical Sciences(CoMS), Department of Emergency Medicine, Bharatpur, Kathmandu, Province-3, Nepal

**Keywords:** Obturator hernia, Abdominal pain, Bowel obstruction, Laparotomy, Howship-Romberg sign, Case report

## Abstract

**Introduction and importance:**

Obturator hernia is a rare abdominal wall hernia (<1 % incidence) that occurs through the obturator foramen, often in elderly, emaciated women. Contrast-enhanced computed tomography (CECT) of the abdomen and pelvis is the diagnostic modality of choice, with a high accuracy of 78 %–100 %.

**Case presentation:**

An 84-year-old frail woman with COPD presented with generalized abdominal pain, abdominal distention, vomiting, and right thigh pain. CECT revealed a right-sided obturator hernia causing small bowel obstruction. Emergency exploratory laparotomy revealed a right-sided strangulated obturator hernia. Postoperatively, the patient developed septic shock and multiorgan dysfunction syndrome (MODS) and succumbed to death on the 5th postoperative day.

**Clinical discussion:**

Obturator hernia is a rare abdominal hernia with an incidence of 0.07–1 %, an often-overlooked condition, more common in elderly women (around 70–90 years) with risk factors like low BMI, multiparity, and chronic conditions such as COPD. It presents with nonspecific symptoms, including abdominal pain, distension, and vomiting, and is often difficult to diagnose. Early CECT has improved the preoperative diagnosis rate from 43 % to 90 %, thus playing a crucial role in preventing morbidity and mortality. Treatment is surgical, but the mortality rate is high due to delayed diagnosis, bowel strangulation, and underlying preexisting illness.

**Conclusion:**

Obturator hernias are a rare but important cause of small bowel obstruction, especially in elderly, frail, malnourished women without prior abdominal surgeries. Medial thigh pain and mild abdominal distension warrant high suspicion and prompt diagnosis using CECT. Early surgical intervention is critical to prevent severe complications and reduce associated morbidity and mortality.

## Highlights

• An **obturator hernia** is a rare abdominal wall hernia (0.07-1% of cases) seen often in elderly, emaciated women.
• Risk **factors** include advanced age, low body mass index, multiparity, and chronic conditions.
• CECT **of the abdomen** is the gold-standard imaging modality for diagnosis, with diagnostic accuracy ranging from 78% to 100%.
• **Surgery is the mainstay of** treatment; the choice of approach (open or laparoscopic) depends on the clinical situation.
• Obturator hernias have high mortality (12-70 %) due to delayed diagnosis, strangulated bowel, and comorbidities in elderly patients.

## 1 Introduction

An obturator hernia is a rare abdominal wall hernia with an incidence of <1 %. It occurs when the abdominal contents protrude through a defect in the obturator foramen and into the obturator canal [1]. It often occurs in elderly, emaciated, and chronically ill women (little old lady's hernia) with non-specific signs of acute intestinal obstruction at presentation [2]. Obturator hernias are potentially the most lethal of all abdominal wall hernias due to their delayed diagnosis and supervening complications of bowel strangulation, perforation, and peritonitis [1]. Contrast-enhanced computed tomography (CECT) of the abdomen and pelvis is the best imaging modality for diagnosis, with its diagnostic accuracy of 78 %–100 % [3]. We are reporting a rare case of an 84-year-old female with abdominal pain associated with abdominal distension, vomiting, and medial thigh pain. The clinical diagnosis of an obstructed right obturator hernia was made with the CECT abdomen and pelvis, and exploratory laparotomy was performed. Intraoperatively a completely transected ileum within the hernial sac was found. This case report has been reported according to the revised SCARE guidelines, 2023 [4].

## 2 Case presentation

**Fig. 1:**
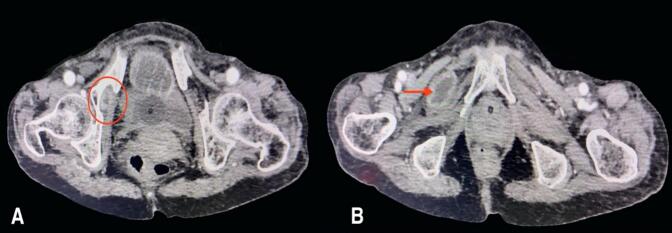
CECT Axial section- A: Small bowel herniating through the right obturator canal. B: Small bowel loop trapped between obturator externus and pectineus muscle. CECT: Contrast-enhanced computed tomography.

**Fig. 2:**
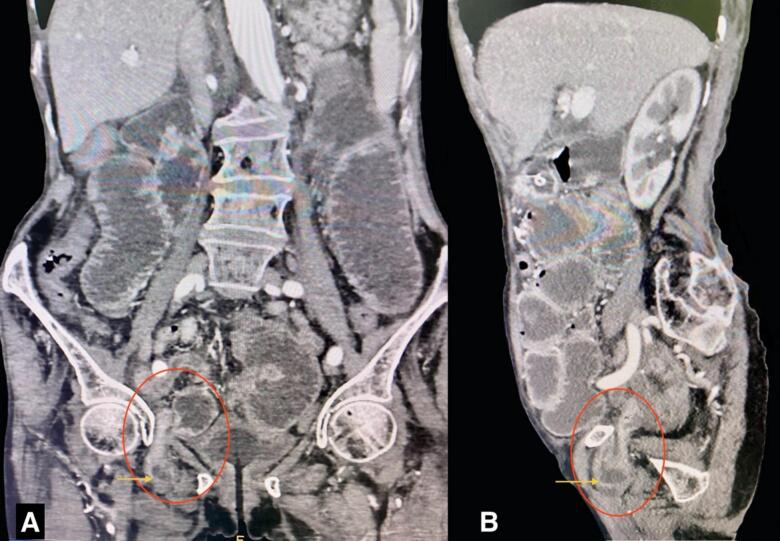
CECT coronal(A) and sagittal(B) section showing right-sided obturator hernia with small bowel (yellow arrow) herniating through the right obturator canal. CECT: Contrast-enhanced computed tomography. (For interpretation of the references to colour in this figure legend, the reader is referred to the web version of this article.)

**Fig. 3:**
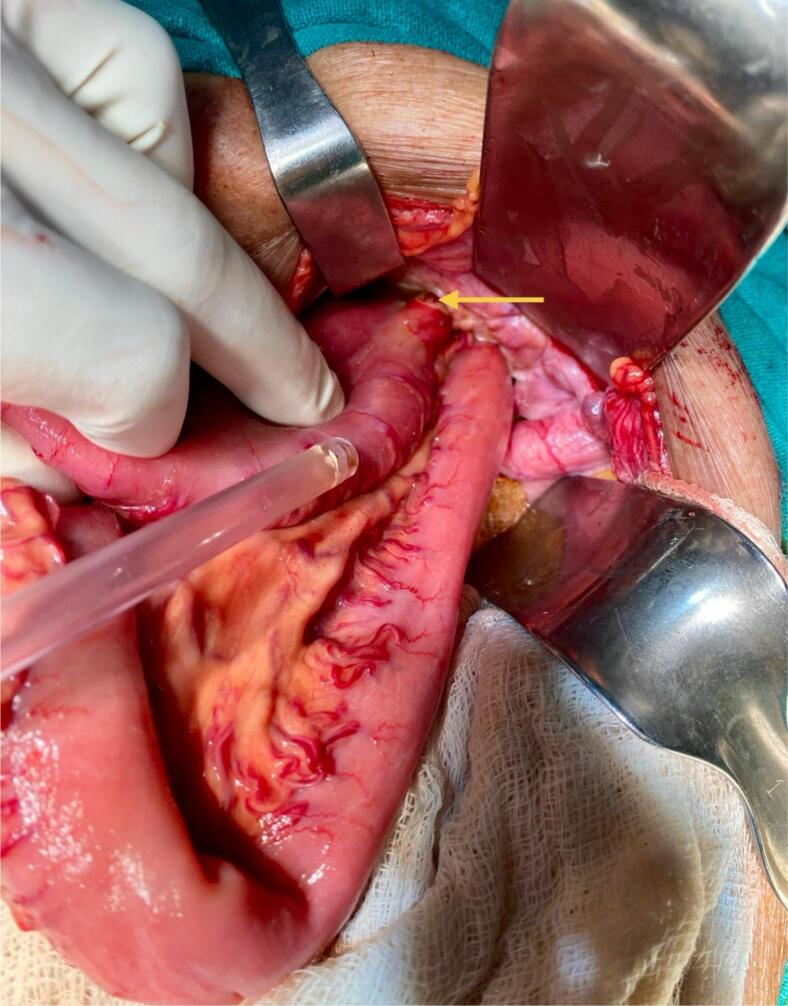
Intraoperative picture showing proximal and distal ileal loop herniating through the right obturator canal. Yellow arrow showing perforated ileum. (For interpretation of the references to colour in this figure legend, the reader is referred to the web version of this article.)

**Fig. 4:**
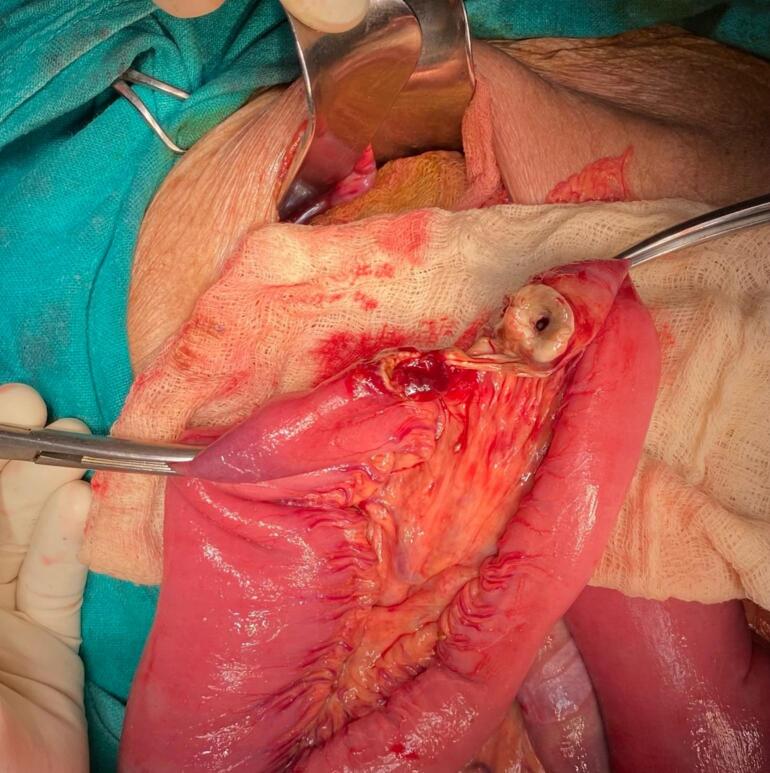
Intraoperative picture showing transected ileum after ileal loop release from obturator canal.

**Fig. 5:**
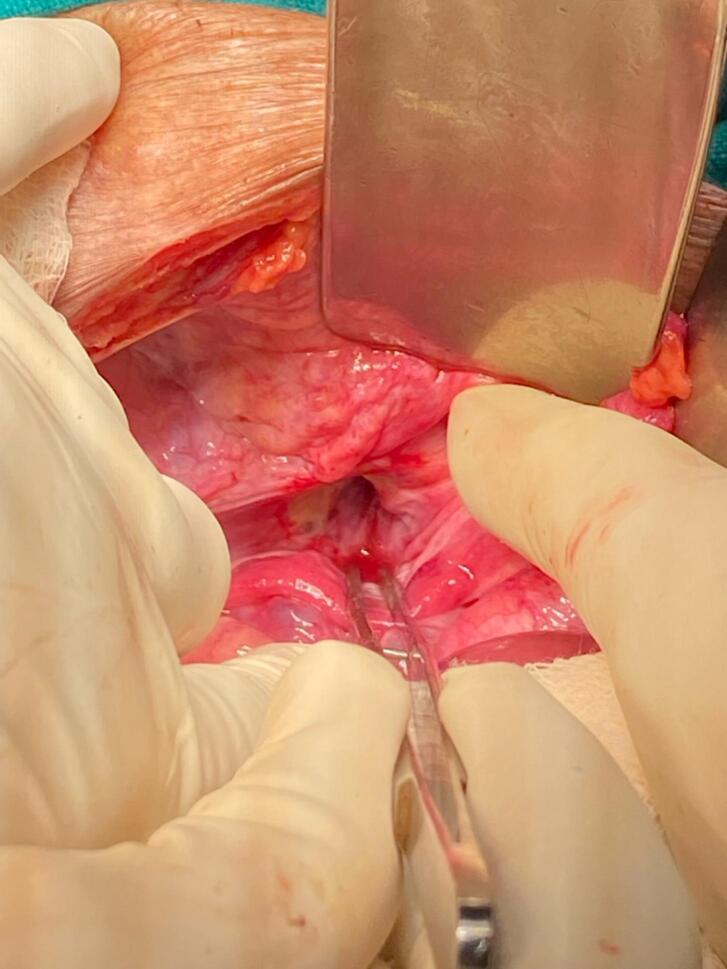
Intraoperative picture showing Obturator foramen.

An 84-year-old woman with chronic obstructive pulmonary disease (COPD) arrived at the emergency department of our hospital complaining of generalized abdominal pain with abdominal distention for 9 days, accompanied by an inability to pass stool and flatus for 4 days and multiple episodes of vomiting for 3 days. The patient also complained of pain in the medial aspect of the right thigh. The patient had a similar history 2 years back, which resolved on its own. The patient was diagnosed with pulmonary tuberculosis 20 years back and completed antitubercular therapy. Besides that, there was no significant medical history. The patient has had 4 pregnancies that were born via normal vaginal delivery in the past. On physical examination, the patient was frail with a BMI of 17.8 kg/m^2^, tachycardic with a pulse rate of 112 beats per minute; the rest of the vitals were within normal limits. There was generalized tenderness all over the abdomen on palpation; however, no rebound tenderness, guarding, or rigidity were elicited. Bowel sounds were sluggish. All hernial orifices were intact. Laboratory parameters were a total leukocyte count of 11,250/cumm with potassium of 2.4 mmol/dL. The rest of the parameters were within the normal limit. Abdominal X-ray erect view showed multiple air-fluid levels with dilated small bowel loops. CECT was done to further investigate the cause of small bowel obstruction, which revealed dilated small bowel loops that can be tracked up to the right pelvis. A loop of small bowel protruding through the obturator foramen with entrapment of the bowel between the obturator externus and pectineus (Fig. 1 and Fig. 2) can be noted, revealing a right-sided obturator hernia. A provisional diagnosis of small bowel obstruction secondary to a right-sided obturator hernia was made. Preoperative stabilization with electrolyte correction with adequate resuscitation was done. The surgical approach chosen for this patient was an emergency exploratory laparotomy via a lower midline incision. This approach was selected due to the emergent nature of the case, providing sufficient access to the right obturator foramen and allowing for the resection of the gangrenous ileal loop (given a long history of symptoms in this patient) and closure of the hernial defect. Intraoperatively, approximately 100 ml of the serous collection was noted in the peritoneal cavity. The ileal loop—150 cm proximal from the ileocecal junction—was strangulated into the right obturator foramen with upstream dilatation of proximal small bowel loops (Fig. 3). On releasing the adhesion and reducing the content from the obturator canal, a complete transection of gangrenous ileal loops (approximately 3 cm of the ileum was gangrenous) was noted (Fig. 4). The gangrenous ileal loop was resected, the proximal dilated small bowel was decompressed, and side-to-side stapled ileal anastomosis was performed. The defect of the obturator foramen (Fig. 5) was closed with an interrupted polypropylene suture using omentum as a patch. Postoperatively, the patient was shifted to the intensive care unit for monitoring. On the 3rd POD, the urine output decreased, TLC raised to 27,000/cumm, and the BP dropped to 80/60 mmHg with a MAP at a range of 60–65. Inotropic support was started, and the patient was monitored closely. Despite intensive monitoring, the patient developed septic shock with multiorgan dysfunction syndrome (MODS) and succumbed to death on the 5th POD.

## 3 Discussion

An obturator hernia is a rare type of abdominal hernia, accounting for 0.07 to 1 % of cases [5]. It occurs when abdominal contents protrude through the obturator canal between the obturator and pectineus muscle [6]. The hernial sac usually contains the small bowel but may also contain the omentum, appendix, bladder, or fallopian tubes [7]. The obturator foramen is present in the iliac bone and is sealed by a thick membrane called an obturator membrane. It is drilled by the obturator foramen, forming the obturator canal (2–3 cm in length and 1 cm in width), which is traversed by obturator vessels and nerves [8]. It is more common in women (6–9 times) due to their broader triangular pelvis, obliquely placed in position and greater transverse diameter [6]. It commonly occurs on the right side (as in our case) because the left one is protected by a sigmoid colon [9]. It affects women of around 70–90 years of age due to atrophy of the preperitoneal fat around the obturator vessels in the canal, thereby predisposing hernia formation; hence it is also called a ‘little old lady's hernia’ [10]. Risk factors for obturator hernia include advanced age (84 years in our case), low body mass index (17.8 in our case), multiparity (Para 4 in our case), and increased intrabdominal pressure due to chronic conditions such as COPD, chronic constipation, and ascites (COPD in our case) [1,11]. Diagnosis of an obturator hernia is very difficult as it usually presents with non-specific signs and symptoms. It is one of the rare causes of intestinal obstruction and occurs in 0.2 %–1.6 % of cases [12]. Major clinical symptoms are due to intestinal obstruction, like abdominal pain, distension, vomiting, and constipation (as in our case), and occur in >80 % of cases of obturator hernia at the time of presentation [13]. History of intermittent previous episodes in the past (seen in our case) is seen in 33 % of cases with or without a palpable mass in the groin [12]. The patient may present with obturator neuralgia characterized by pain in the medial thigh that worsens with adduction, extension, and medial rotation of the thigh (Howship-Romberg sign) due to compression of the obturator nerve's cutaneous branch and is observed in <50 % of cases [14]. The Hannington-Kiff sign, on the other hand, is an absent adductor reflex in the thigh resulting from obturator nerve compression [15]. CECT of the abdomen and pelvis is the first-choice imaging modality for the diagnosis of obturator hernia with its diagnostic accuracy and specificity of 78 %–100 % [3]. It can show the herniated content extending through the obturator foramen between the pectineus and obturator externus muscles. In incarcerated hernia, dilated proximal bowel loops and enhancement patterns of herniated bowel loops can also be seen. It can also detect asymptomatic bilateral obturator hernias [16]. Since the use of CT scans, the preoperative diagnosis rate has improved from 43 % to 90 % in some reports. Diagnostic laparoscopy serves as a tool in diagnosing and treating occult, suspected, and missed obturator hernias on imaging [17]. The only treatment for obturator hernia is surgery. The retropubic and transperitoneal inguinal approaches, low midline incision laparotomy, and the growing preference for laparoscopic surgery are among the favored surgical methods in current practice [18]. The laparoscopic approach may be more beneficial for elderly patients due to fewer postoperative complications. In an emergency, the abdominal open technique with a low midline incision is most frequently used because it provides sufficient exposure to the obturator ring and permits the resection of any ischemic bowel. Simple closure of the defect with interrupted non-absorbable suture leaving behind the sac can be done in the setting of perforation and gangrenous bowel. In our case, we also closed the defect of the obturator foramen with an interrupted polypropylene suture. (The decision to perform an open laparotomy in our case was made due to the emergent nature of the surgery and the need for extensive exploration). It has an acceptable recurrence rate of <10 % [19]. Despite being a rare type of abdominal hernia, the mortality rate is notably high, ranging from 12 % to 70 %. This elevated rate is attributed to several factors, including reduced physiological reserves in elderly patients, exacerbated by emaciation, malnutrition, and significant weight loss. Additionally, pre-existing medical conditions complicate perioperative care, while a higher likelihood of bowel strangulation and delays in diagnosis and treatment due to subtle symptoms further contribute to the increased mortality [20]. In our case, an old, emaciated female with preexisting medical illness (COPD) and a longer duration of symptoms (9 days) led to strangulated ileum and ultimately unfavorable postoperative outcomes. Post-operative management in high-risk patients, like the one in this case of strangulated obturator hernia, involves aggressive supportive care to address complications such as septic shock and multiorgan dysfunction syndrome (MODS). Key strategies include early identification and management of infections, maintaining hemodynamic stability with IV fluids and inotropes, close monitoring in the ICU, and addressing electrolyte imbalances (e.g., hypokalemia). Early nutritional support, infection control, and respiratory management are essential in frail patients, especially those with COPD, to minimize further complications. Mortality can be high due to underlying conditions, delayed diagnosis, and bowel ischemia [21,22]. The key clinical takeaway from this case is the importance of considering obturator hernia in elderly patients presenting with small bowel obstruction, particularly when accompanied by medial thigh pain. In this case, CECT enabled timely diagnosis of the strangulated hernia. Despite successful surgery, the patient developed septic shock and MODS, ultimately leading to her death. This highlights the difficulties in managing elderly patients with significant comorbidities and underscores the need for aggressive postoperative care, including hemodynamic support, infection monitoring, and early nutritional intervention [22].

## 4 Conclusion

Obturator hernias, though rare, are a significant cause of small bowel obstruction, particularly in elderly, frail, and malnourished female patients with no prior abdominal surgeries. Clinicians should maintain a high index of suspicion in cases presenting with medial thigh pain (Howship-Romberg sign), pain abdomen, and abdominal distension. Prompt diagnosis through CECT of the abdomen and pelvis is crucial for early identification. Early surgical intervention is essential to reduce the high morbidity and mortality associated with delayed diagnosis and complications such as bowel ischemia, necrosis, and perforation.

## CRediT authorship contribution statement

Constructing hypothesis for the manuscript- Samrat Shrestha, Sabin K Ghimire.

Planning methodology to reach the conclusion: Sabin K Ghimire, Samrat Shrestha, Suresh Maharjan.

Organizing and supervising the course of the article and taking responsibility: Samrat Shrestha

Patient follow-up and reporting –Rahul Jha, Mecklina Shrestha, Suresh Maharjan.

Logical interpretation and presentation of the results- Samrat Shrestha, Sabin K Ghimire, Rahul Jha, Suresh Maharjan, Mecklina Shrestha.

Construction of the whole or body of the manuscript- Samrat Shrestha, Sabin K Ghimire, Mecklina Shrestha, Rahul Jha, Suresh Maharjan.

Reviewing the article before submission not only for spelling and grammar but also for its intellectual content- Samrat Shrestha, Sabin K Ghimire, Rahul Jha, Suresh Maharjan, Mecklina Shrestha.

## Consent

Written informed consent was taken from the patient who participated in this study for publication of this case report and accompanying images.

## Ethical approval

The IRB at our institution has waived ethical approval for case reports.

## Guarantor

The guarantor is Samrat Shrestha.

## Funding

There are no sources of funding for this case study to declare.

## Registration of research studies

None.

## Declaration of competing interest

The authors have no conflict of interest to declare.
